# Targeting Vascular and Inflammatory Crosstalk: Cannabigerol as a Dual-Pathway Modulator in Rosacea

**DOI:** 10.3390/ijms26146840

**Published:** 2025-07-16

**Authors:** Suji Kim, Ji Hyun Lee

**Affiliations:** 1 Department of Medical Sciences, Graduate School of The Catholic University of Korea, Seoul 06591, Republic of Korea; suezie0809@gmail.com; 2 Department of Dermatology, Seoul St. Mary’s Hospital, College of Medicine, The Catholic University of Korea, Seoul 06591, Republic of Korea

**Keywords:** cannabigerol, rosacea, inflammation, angiogenesis, JAK/STAT signaling, yes-associated protein (YAP), transcription activator with a PDZ-binding motif (TAZ)

## Abstract

Rosacea is a chronic inflammatory skin condition characterized by persistent erythema and abnormal vascular response. Although current treatments focus on symptomatic relief, they often provide only temporary improvement and may be associated with side effects or recurrence. Cannabigerol (CBG), a non-psychoactive cannabinoid, has recently garnered attention for its pharmacological activities, including anti-inflammatory, antioxidant, neuroprotective, and skin barrier–supportive effects. However, its role in modulating pathological responses in rosacea remains unclear. In this study, we investigated the therapeutic potential of topically applied CBG in an LL-37-induced rosacea-like mouse model. Clinical and histological assessments revealed that CBG markedly reduced erythema, epidermal hyperplasia, and mast cell infiltration. Quantitative reverse transcription polymerase chain reaction (qRT-PCR) showed downregulation of *Il1b*, *Il4*, *Il6*, *Il13*, *Il22*, *Il31*, *Tlr2*, *Vegfa*, and *Mmp9*. Immunohistochemistry and Western blot analyses further demonstrated suppression of CD31, vascular endothelial growth factor (VEGF), and Yes-associated protein (YAP) and transcriptional coactivator with PDZ-binding motif (TAZ), along with reduced activation of the Janus kinase/signal transducer and activator of transcription (JAK/STAT) pathway, including decreased levels of JAK1, STAT3, and phosphorylated STAT3. These findings suggest that topical CBG alleviates rosacea-like skin inflammation by targeting inflammatory and vascular pathways, including JAK/STAT and YAP/TAZ signaling.

## 1. Introduction

Rosacea is a chronic inflammatory skin disorder characterized by persistent facial erythema, telangiectasia, flushing, papules, and pustules, most commonly affecting the central areas of the face, including the nose, cheeks, and forehead [1,2,3]. Among these, erythema and telangiectasia are the most prominent clinical features and are frequently exacerbated by external stimuli such as ultraviolet radiation and temperature fluctuations [4]. Although the precise pathogenesis of rosacea has not yet been fully elucidated, it is generally considered to result from a complex interplay between immune dysregulation, neurovascular imbalance, and microbial dysbiosis in the skin [5,6].

Current therapeutic strategies primarily aim at symptomatic relief through the use of topical and systemic agents, including antibiotics, retinoids, and anti-inflammatory drugs [7]. In addition, laser- and light-based therapies targeting erythema and telangiectasia have shown clinical effectiveness [8]. However, these approaches often provide only temporary improvements and are associated with side effects with long-term use or relapse after discontinuation [9]. Consequently, the management of rosacea remains challenging, underscoring the need for more effective, durable, and personalized treatments based on clinical phenotypes.

Cannabigerol (CBG), a non-psychoactive cannabinoid, has recently gained increasing attention as a potential therapeutic agent for various inflammatory and pain-related disorders [10,11]. Preclinical studies have shown that CBG alleviates inflammation and fibrosis in models of non-alcoholic steatohepatitis, modulates the cutaneous endocannabinoid system, and reduces disease severity in experimental models of inflammatory bowel disease, arthritis, and neuropathic pain [11,12]. In our previous study using a murine model of atopic dermatitis, topical application of CBG significantly reduced skin inflammation and improved epidermal barrier function [13]. Collectively, these findings suggest that CBG may offer therapeutic benefits in rosacea, a chronic inflammatory skin condition, with limited long-term treatment options.

Vascular dysfunction plays a central role in rosacea pathophysiology. Abnormal reactivity of cutaneous blood vessels and endothelial cell irregularities directly contribute to persistent erythema and telangiectasia [14]. Inflammatory mediators, such as matrix metalloproteinases (MMPs) and vascular endothelial growth factor (VEGF), promote angiogenesis and vascular remodeling, further exacerbating the condition [3]. In addition, mast cells, frequently activated by LL-37, have been reported to contribute to the pathogenesis of rosacea by releasing pro-inflammatory and pro-angiogenic mediators, including VEGF, thus linking innate immune responses to vascular abnormalities [15,16]. Recent studies have highlighted that vascular dysfunction is not merely a secondary manifestation of rosacea but may actively contribute to disease progression [17,18]. Pro-angiogenic factors, such as VEGFA, are upregulated in rosacea lesions and promote neovascularization and vasodilation, worsening the clinical erythema [19]. Yes-associated protein (YAP) and transcriptional coactivator with PDZ-binding motif (TAZ), which are mechanosensitive transcriptional coactivators, have been shown to play key roles in dermal vascular remodeling during chronic skin inflammation [20]. In this context, the YAP/TAZ signaling pathway has emerged as a critical regulator of VEGFA transcription in response to mechanical and inflammatory stimuli [21,22].

Meanwhile, the Janus kinase/signal transducer and activator of transcription (JAK/STAT) signaling pathway, particularly the JAK1/STAT3 axis, has been implicated in both immune activation and vascular regulation in inflammatory skin diseases [23]. Cytokines such as IL-6 and IL-22 activate STAT3, which in turn promotes the transcription of genes involved in inflammation, angiogenesis, and endothelial proliferation, including VEGFA and IL-1β [19,20]. Given their central involvement in the pathogenesis of rosacea, both YAP/TAZ and JAK/STAT signaling are promising therapeutic targets.

In the present study, we investigated the therapeutic potential of CBG in rosacea, focusing on its ability to modulate the JAK/STAT and YAP/TAZ signaling pathways. By evaluating its effects on both inflammatory and vascular components of the disease, we aimed to elucidate the potential of CBG as a novel and promising treatment option for rosacea.

## 2. Results

### 2.1. Cannabigerol Alleviates Redness and Inflammation in LL-37-Induced Rosacea Mouse Model

**Figure 1:**
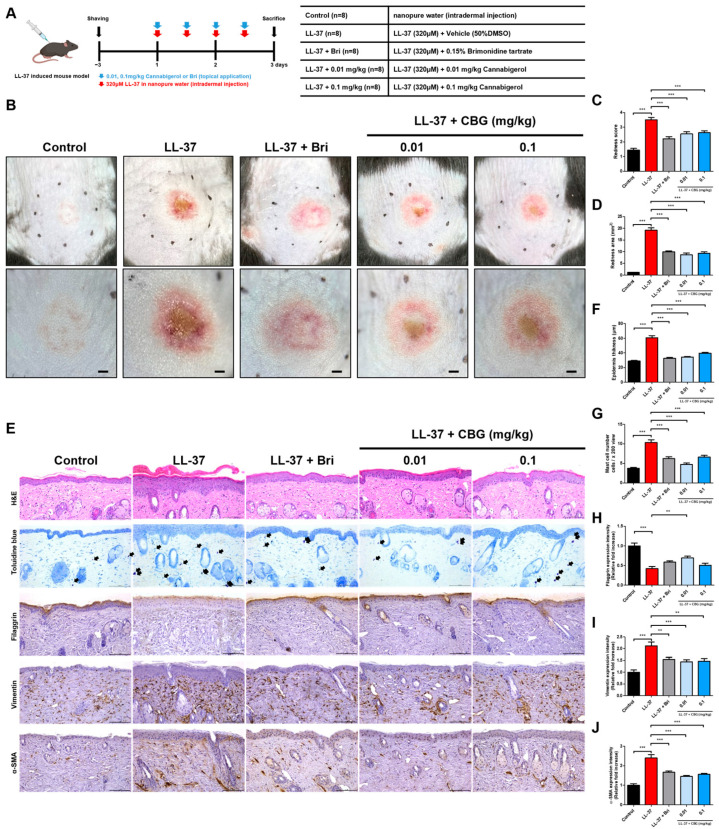
Clinical and histological evaluation of rosacea-like inflammation after topical CBG treatment. (**A**) Experimental schedule and group allocation in LL-37-induced rosacea-like mouse model (*n* = 8/group). (**B**) Representative clinical images of the dorsal skin taken after sacrifice. Upper panel: captured by camera; lower panel: dermoscopic view. Scale bars = 1 mm. (**C**) Redness score was assessed based on clinical images by three blinded dermatologists using a 0–4 scale. (**D**) Quantification of the redness area using ImageJ software (version 1.54f). (**E**) Representative mouse skin tissue sections stained with H&E, toluidine blue, and immunohistochemistry for filaggrin, vimentin, and α-SMA. Purple-stained dermal mast cells are shown. Scale bar = 100 μm (200× magnification; arrows indicate mast cells). (**F**) Quantitative analysis of epidermis thickness from H&E-stained sections. (**G**) Quantification of mast cell numbers based on toluidine blue staining. (**H**–**J**) Quantitative analysis of IHC staining for filaggrin (**H**), vimentin (**I**), and α-SMA (**J**). All data are shown as the mean ± SEM (*n* = 8). Statistical analysis was performed using one-way ANOVA ** *p* < 0.01, *** *p* < 0.001 compared to the control or LL-37 groups. Bri, brimonidine; LL-37, cathelicidin peptide LL-37; CBG, cannabigerol; DMSO, dimethyl sulfoxide; α-SMA, alpha-smooth muscle actin; IHC, Immunohistochemical; H&E, hematoxylin and eosin.

To investigate the therapeutic effects of CBG in rosacea, we established a rosacea-like model by subcutaneous injection of LL-37 in C57BL/6 mice. A total of 40 eight-week-old mice were divided into five groups (*n* = 8): Control, LL-37, LL-37 + brimonidine (Bri), LL-37 + CBG (0.01 or 0.1 mg/kg). LL-37 (320 μM) was injected subcutaneously four times at 12 h intervals over two days, with topical treatments applied immediately after each injection. Skin samples were harvested the following day (Figure 1A). LL-37 injection induced a pronounced increase in erythema compared to the control group, confirming successful model induction (*p* < 0.001; Figure 1C). Topical CBG application significantly reduced redness scores, with the 0.01 mg/kg group showing the most notable improvement compared to the LL-37 group (*p* < 0.001; Figure 1C). Quantitative analysis of the redness area further supported these results, demonstrating a significant reduction in erythema in both CBG-treated groups, with a lower dose being more effective (*p* < 0.001; Figure 1D).

Histological examination revealed that CBG treatment significantly reduced dermal inflammatory cell infiltration, particularly mast cells (*p* < 0.001; Figure 1G), with more pronounced effects at 0.01 mg/kg. Moreover, CBG markedly attenuated epidermal thickening (*p* < 0.001; Figure 1F), which further supports its anti-inflammatory effects on skin architecture. To extend these findings and assess skin remodeling at the molecular level, we examined the immunohistochemical expression of filaggrin, vimentin, and α-SMA (Figure 1E). LL-37 injection markedly decreased filaggrin expression while increasing vimentin and α-SMA levels, indicating epidermal barrier disruption and dermal fibroblast activation (*p* < 0.001; Figure 1H–J). Topical CBG treatment significantly increased filaggrin levels in the 0.01 mg/kg group compared to the LL-37 group (*p* < 0.01; Figure 1H). Vimentin expression was reduced in both CBG-treated groups, with stronger significance in the 0.01 mg/kg group (*p* < 0.001) and moderate significance in the 0.1 mg/kg group (*p* < 0.01; Figure 1I). α-SMA levels were significantly decreased in both CBG groups (*p* < 0.001; Figure 1J), reinforcing the observed improvements in dermal inflammation and tissue remodeling.

### 2.2. Cannabigerol Suppresses Inflammatory and Angiogenic Gene Expression

**Figure 2:**
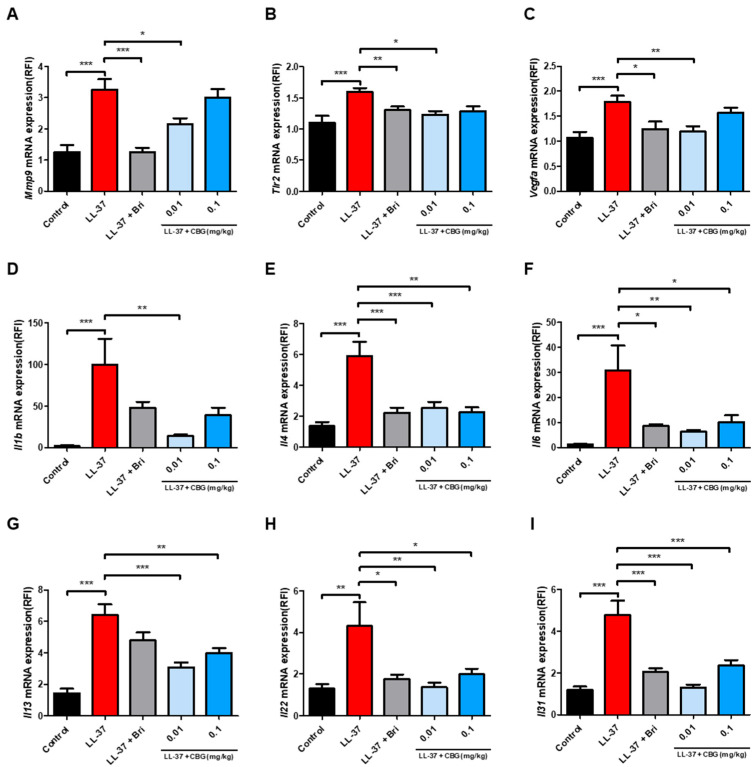
Topical CBG treatment attenuated the expression of inflammation- and angiogenesis-related genes in LL-37-induced rosacea-like mouse skin. Quantitative real-time PCR (qPCR) analysis of mRNA expression levels of (**A**) *Mmp9*, (**B**) *Tlr2*, (**C**) *Vegfa*, (**D**) *Il1b*, (**E**) *Il4*, (**F**) *Il6*, (**G**) *Il13*, (**H**) *Il22*, and (**I**) *Il31* in the lesional dorsal skin. Gene expression was normalized to that of *Actb* and expressed relative to that of the control group. Data are presented as mean ± SEM (*n* = 8). Results represent three independent experiments. Statistical significance was assessed using one-way ANOVA * *p* < 0.05, ** *p* < 0.01, and *** *p* < 0.001 compared to the control and LL-37 groups. Bri, brimonidine; CBG, cannabigerol; TLR2, Toll-like receptor 2; MMP9, matrix metalloproteinase-9; VEGF, vascular endothelial growth factor; IL, interleukin; LL-37, cathelicidin peptide LL-37.

To assess molecular responses to CBG, we performed qRT-PCR analysis on dorsal skin tissues. LL-37 significantly upregulated Toll-like receptor 2 (*Tlr2*) and several pro-inflammatory cytokines, including *Il1β*, *Il4*, *Il6*, *Il13*, *Il22*, and *Il31*, compared to the controls (*p* < 0.001; Figure 2B,D–I). Topical application of CBG at 0.01 mg/kg markedly downregulated *Tlr2* (*p* < 0.05), *Il1b* (*p* < 0.01), *Il4* (*p* < 0.001), *Il6* (*p* < 0.01), *Il13* (*p* < 0.001), *Il22* (*p* < 0.01), and *Il31* (*p* < 0.001) expression levels. In the 0.1 mg/kg group *Il4* (*p* < 0.01), *Il6* (*p* < 0.05), *Il13* (*p* < 0.01), *Il22* (*p* < 0.05), and *Il31* (*p* < 0.001) were also significantly decreased, whereas *Tlr2* and *Il1b* showed a downward trend without statistical significance.

In parallel, LL-37 induced substantial increases in *Mmp9* and *Vegfa*, both related to tissue remodeling and angiogenesis (*p* < 0.001; Figure 2A,C). The 0.01 mg/kg CBG group led to significant downregulation of *Mmp9* (*p* < 0.05) and *Vegfa* (*p* < 0.01), while the higher dose exhibited a mild reduction.

These results suggest that topical CBG effectively suppresses both inflammatory signaling and pro-angiogenic gene expression in rosacea-like skin, with more consistent outcomes at lower concentrations.

### 2.3. Cannabigerol Inhibits Vascular Activation and YAP/TAZ Signaling Pathway

**Figure 3:**
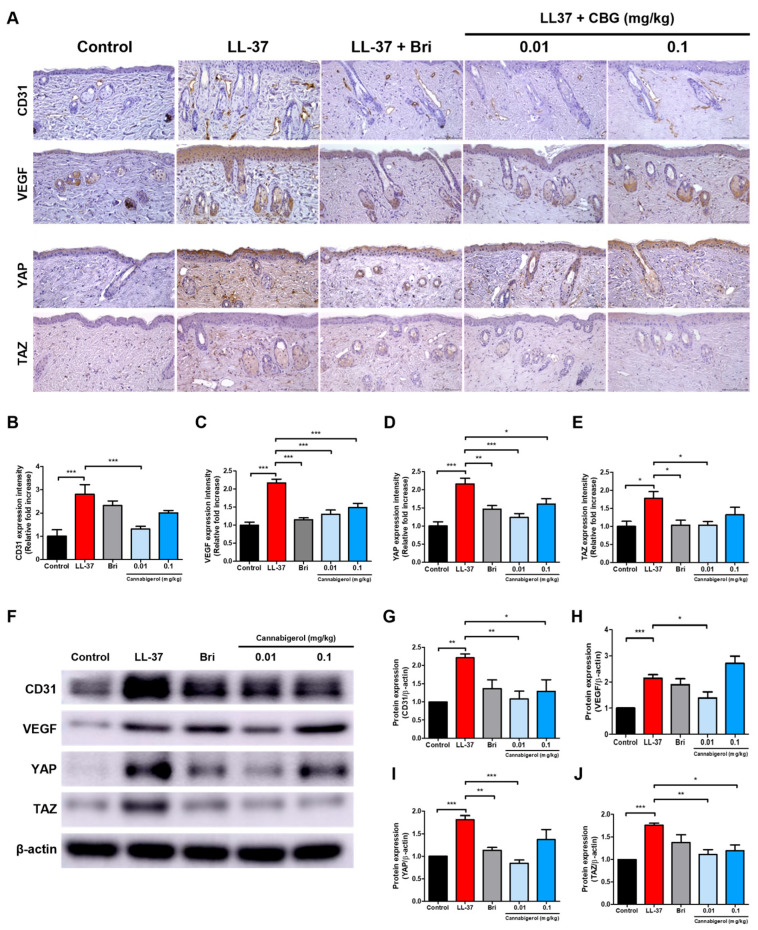
Treatment with CBG reduces vascular markers and YAP/TAZ protein expression in LL-37-induced rosacea-like skin lesions. (**A**) Immunohistochemical (IHC) staining of CD31, VEGF, YAP, and TAZ in dorsal skin sections (original magnification, ×200; scale bar = 100 μm). (**B**–**E**) Quantification of IHC staining intensity using Fiji ImageJ software. (**F**) Representative Western blot images showing the protein expression levels of CD31, VEGF, YAP, and TAZ in dorsal skin lysates. (**G**–**J**) Quantitative densitometric analysis of protein bands normalized to β-actin using the ImageJ software (version 1.54f). Data are presented as mean ± SEM (*n* = 8). * *p* < 0.05, ** *p* < 0.01, and *** *p* < 0.001 compared with the control or LL-37 groups. CBG, cannabigerol; Bri, brimonidine; VEGF, vascular endothelial growth factor; YAP, yes-associated protein; TAZ, transcriptional coactivator with PDZ-binding motif; LL-37, cathelicidin peptide LL-37.

To investigate the effect of CBG on angiogenesis and mechanical signaling, we examined CD31, VEGF, YAP, and TAZ expression in the lesional skin. Immunohistochemical staining was performed to assess the expression of CD31, VEGF, YAP, and TAZ in the dermis (Figure 3A). LL-37 injection markedly increased the dermal expression of all four markers (*p* < 0.001; Figure 3B–E). In contrast, CBG treatment, especially at 0.01 mg/kg, significantly reduced CD31 and VEGF expression (*p* < 0.001; Figure 3B,C) as well as YAP and TAZ levels (*p* < 0.001 and *p* < 0.05; Figure 3D,E). At 0.1 mg/kg, CBG also significantly decreased VEGF and YAP expression (*p* < 0.001 and *p* < 0.05; Figure 3C,D), whereas reductions in CD31 and TAZ were not statistically significant. To confirm these results at the protein level, Western blotting was conducted for CD31, VEGF, YAP, and TAZ (Figure 3F). Quantitative analysis confirmed that CBG treatment significantly decreased the protein expression of these markers, particularly at 0.01 mg/kg, including CD31 and TAZ (*p* < 0.01; Figure 3G,J), VEGF (*p* < 0.05; Figure 3H), and YAP (*p* < 0.001; Figure 3I). Additionally, 0.1 mg/kg CBG also significantly reduced CD31 and TAZ expression (*p* < 0.05; Figure 3G,J). Actin served as the internal control for normalization.

### 2.4. Cannabigerol Inhibits JAK/STAT Signaling Pathway

**Figure 4:**
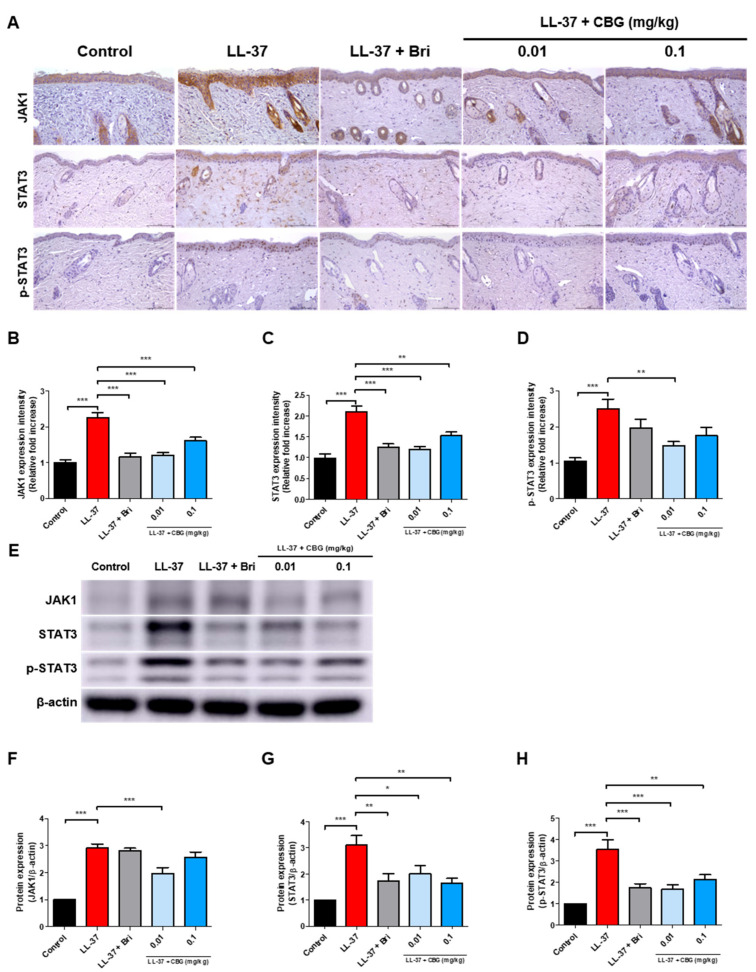
CBG inhibits JAK–STAT pathway activation in LL-37-induced rosacea-like skin lesions. (**A**) Immunohistochemical (IHC) staining of JAK1, STAT3, and phosphorylated STAT3 (p-STAT3) in dorsal skin sections (original magnification, ×200; scale bar = 100 μm). (**B**–**D**) Quantification of IHC staining intensity using Fiji ImageJ software. (**E**) Representative Western blot images of JAK1, STAT3, and p-STAT3 protein expression in the dorsal skin lysates. (**F**–**H**) Quantitative densitometric analysis of Western blot bands normalized to actin using the ImageJ software. Data are presented as mean ± SEM (*n* = 8). * *p* < 0.05, ** *p* < 0.01, and *** *p* < 0.001 compared to the control or LL-37 group. CBG, cannabigerol; Bri, brimonidine; JAK, Janus kinase; STAT, signal transducer and activator of transcription; p-STAT3, phosphorylated STAT3; LL-37, cathelicidin peptide LL-37.

Immunohistochemical staining was performed to assess dermal expression of JAK1, total STAT3, and phosphorylated STAT3 (p-STAT3) (Figure 4A). LL-37 injection significantly increased the expression of JAK1, total STAT3, and p-STAT3 (*p* < 0.001; Figure 4B–D). Topical treatment with CBG significantly reduced JAK1 and STAT3 expression levels at both 0.01 and 0.1 mg/kg doses with the most pronounced effects observed at 0.01 mg/kg (*p* < 0.001 and *p* < 0.01; Figure 4B,C). In contrast, p-STAT3 was significantly reduced only to 0.01 mg/kg (*p* < 0.01; Figure 4D). Protein level confirmation via Western blot analysis is shown in Figure 4E. Quantitative densitometry indicated that CBG treatment at 0.01 mg/kg significantly reduced the protein expression of JAK1 and p-STAT3 (*p* < 0.001; Figure 4F,H), and moderately decreased total STAT3 levels (*p* < 0.05; Figure 4G). At 0.1 mg/kg CBG also significantly suppressed STAT3 and p-STAT3 expression (*p* < 0.01; Figure 4G,H), while having minimal effect on JAK1. These findings suggest that CBG effectively inhibits JAK1-mediated activation of STAT3 in rosacea-like skin. Taken together, the findings indicate that CBG suppresses inflammatory signaling via inhibition of the JAK/STAT pathway, thereby enhancing its anti-inflammatory efficacy. These results indicate that topical CBG inhibits angiogenic and mechanosensitive pathways by concurrently reducing mRNA and protein levels of CD31, VEGF, YAP, and TAZ, thereby contributing to the reduction of vascular activation in rosacea-like inflammation.

## 3. Discussion

In this study, we demonstrated for the first time the therapeutic potential of topical CBG using a rosacea-like mouse model induced by LL-37. This model successfully reproduced the characteristic features of rosacea, such as visible erythema, a thickened epidermis, inflammatory infiltration and increased angiogenesis. Following CBG application downregulates the activity of the YAP/TAZ pathway and inhibits JAK/STAT signaling, both of which are upregulated in the disease state. CBG also reduced the expression of vascular and inflammatory markers.

Brimonidine is currently the only FDA-approved topical treatment for persistent facial erythema in rosacea. As an α2-adrenergic receptor agonist, it primarily alleviates redness through vasoconstriction. Previous studies have shown that brimonidine may have limited effects on inflammatory and vascular markers, possibly through indirect modulation of dermal reactivity [24,25]. In contrast, CBG demonstrated broader efficacy by directly modulating inflammatory cytokines, vascular markers, and associated signaling pathways, suggesting its potential as a multifaceted therapeutic agent beyond symptomatic relief.

Phytocannabinoids have attracted growing interest as therapeutic agents for inflammatory skin diseases, largely due to their ability to modulate immune responses, restore barrier function, and suppress cutaneous inflammation [26]. Among these, cannabidiol (CBD) has been the most extensively studied, with numerous reports demonstrating its clinical and preclinical efficacy in treating psoriasis, atopic dermatitis, and contact dermatitis [27,28,29]. Our laboratory previously investigated the effects of various phytocannabinoids, primarily focusing on CBD and cannabichromene (CBC), in skin inflammation models [28,30]. CBG, although less studied compared to other cannabinoids, has been shown to exert anti-inflammatory effects in keratinocytes and fibroblasts, which are key epidermal and dermal cell types involved in skin immunity [31]. In previous studies, CBG, applied alone or in combination with CBD, was shown to reduce oxidative and inflammatory stress markers even in the absence of UVA stimulation, suggesting a basal immunomodulatory role [32,33]. Notably, we recently demonstrated that topical application of CBG significantly reduced clinical severity and inflammatory markers in a murine model of atopic dermatitis, while also improving epidermal barrier integrity and reducing mast cell accumulation [13]. Moreover, CBG is known to regulate inflammatory signaling in keratinocytes and fibroblasts, which are key players in rosacea pathophysiology [12]. In addition to these functional benefits, the safety of topical CBG in non-inflamed skin has also been confirmed in previous studies, which reported no significant toxicity or adverse effects [33,34]. Based on this background, we designed the present study to investigate the therapeutic efficacy of CBG in an LL-37-induced rosacea-like inflammation model.

Building on these findings, we evaluated the therapeutic effect of topical CBG in an LL-37-induced rosacea-like inflammation model, in which topical CBG treatment effectively alleviated erythema, epidermal thickening, and inflammatory cell infiltration. Mast cells are known to contribute to rosacea pathophysiology by releasing histamine, matrix metalloproteinases such as MMP9, and various pro-inflammatory cytokines in response to LL-37 stimulation [16]. These mediators promote vasodilation, angiogenesis, and fibrosis, which are key features of rosacea progression [35]. In our study, dermal mast cell numbers were notably increased in the LL-37-induced rosacea-like model, while topical CBG treatment led to a marked reduction in dermal mast cell infiltration, highlighting its potential to suppress mast-cell-mediated inflammation in rosacea.

In this LL-37-induced rosacea-like model, the mRNA expression of several key mediators including *Mmp9* and *Vegfa* was markedly upregulated. These molecules are involved in diverse inflammatory and vascular processes such as immune cell recruitment, epithelial dysregulation, and abnormal angiogenesis, and their elevated expression has been reported in both experimental models and human rosacea lesions [36,37]. Topical CBG treatment significantly suppressed the expression of these mediators, indicating its potential to modulate rosacea-associated inflammatory and vascular pathways.

Recent studies suggest that the therapeutic effects of CBG may involve interactions with multiple molecular targets. CBG has been reported to activate cannabinoid receptor 2 (CB2), which plays a crucial role in suppressing pathological angiogenesis. Activation of CB2 reduces VEGF expression and attenuates endothelial cell activation through inhibition of STAT3 and HIF-1α signaling pathways [38]. These findings suggest that the downregulation of VEGF and phosphorylated STAT3 observed in our study may be partially mediated by CB2 activation.

In addition, CBG has been shown to activate peroxisome proliferator-activated receptor gamma (PPAR-γ), a nuclear receptor involved in immune regulation and cytokine suppression. PPAR-γ activation downregulates IL-6 and inhibits STAT3 phosphorylation, contributing to the suppression of pro-inflammatory signaling [12]. Although our current study did not directly examine CB2 or PPAR-γ activation, the observed reduction in IL-6, VEGF, and p-STAT3 expression aligns with these reported mechanisms. Further investigations will be necessary to confirm these pathways in rosacea models.

Angiogenesis is a key pathological feature of rosacea, contributing to persistent erythema and dermal vascular hyperreactivity. Among the pro-angiogenic mediators, VEGF plays a central role in promoting endothelial proliferation, vascular permeability, and neovascularization [39,40]. Several studies have used CD31 (PECAM-1) as an indicator of dermal vascular density to evaluate VEGF-associated angiogenic activity. Several previous studies have reported VEGF-associated CD31 upregulation in rosacea lesions or post-acne erythema [21,40,41]. Moreover, it has been suggested that VEGF signaling interacts with YAP and TAZ to promote angiogenesis in various diseases. In developmental angiogenesis, VEGF directly activates YAP/TAZ transcriptional programs to coordinate endothelial expansion and vascular formation [22]. In tumor models, VEGFA has been associated with enhanced YAP/TAZ activation via receptor-mediated signaling, contributing to angiogenic switching and tissue invasion [42]. In diabetic retinopathy, YAP/TAZ signaling contributes to VEGF-induced endothelial proliferation and microvascular dysfunction under high-glucose conditions [43]. In our previous study on atopic dermatitis, we also found that dysregulation of the Hippo pathway leads to epidermal thickening and inflammatory amplification, supporting its broader relevance in inflammatory skin disorders [44]. In a retinal angiogenesis model, YAP was reported to interact with STAT3 to promote VEGF-induced endothelial cell actions [45], leading to the suggestion of potential crosstalk between VEGF, YAP/TAZ, and STAT3 signaling pathways in endothelial cells. In the present study, LL-37-induced lesions exhibited increased expression of VEGF, CD31, YAP, and TAZ. Topical CBG treatment markedly suppressed all of these markers, suggesting that CBG interferes with both angiogenic signaling and the transcriptional activation linked to vascular stress. These findings reinforce the potential of CBG to modulate vascular remodeling pathways in rosacea by targeting the VEGF–YAP/TAZ axis.

Meanwhile, the JAK/STAT signaling cascade is a key mediator of cytokine-induced transcriptional responses, integrating immune cell activation, epithelial remodeling, and inflammatory amplification. STAT3, in particular, is activated downstream of multiple pro-inflammatory and Th2-associated cytokines and has been implicated in a wide range of inflammatory skin diseases and tumors [23,46,47]. In rosacea, emerging data point to the involvement of the JAK/STAT pathway in sustaining chronic inflammation and vascular dysfunction. Transcriptomic analyses have identified STAT3 as a central regulatory node in rosacea skin [48], and experimental models have confirmed that LL-37 induces upregulation of JAK1, STAT3, and phosphorylated STAT3 in murine lesions [49,50]. Therapeutic inhibition of this pathway alleviates clinical severity, supporting its pathogenic relevance [51]. Given the documented interactions between STAT3 and other inflammatory pathways such as VEGF and YAP/TAZ in endothelial and epithelial cells [45,52], it is possible that CBG’s suppression of STAT3 contributes to its broader anti-angiogenic and anti-inflammatory effects in rosacea. In this study, topical CBG treatment reduced the increased JAK1 and p-STAT3 expression after LL-37 injection. Our findings support the hypothesis that the JAK/STAT signaling pathway constitutes a key pathogenic loop in rosacea and may serve as a therapeutic target for anti-inflammatory intervention.

Among the cytokines evaluated in our study, *Il1b* and *Il6* are classical mediators of innate immunity. IL-1β promotes leukocyte infiltration and inflammatory cytokine production and has been shown to indirectly activate the STAT3 pathway in keratinocytes and myeloid cells [36,47,53]. IL-6 acts more directly by engaging the IL-6 receptor complex, leading to STAT3 phosphorylation and downstream amplification of inflammatory gene expression. Persistent IL-6–STAT3 signaling has been linked to tissue remodeling and disease persistence in rosacea and psoriasis [23,48,50].

IL-22, a cytokine associated with Th22 immunity, is another potent activator of STAT3. It promotes keratinocyte hyperproliferation, inhibits differentiation, and disrupts barrier integrity through STAT3-dependent transcription of proliferation-related genes [50,54]. Recent reviews have further emphasized the pathogenic implications of IL-22 and STAT3 signaling in keratinocyte-driven inflammation and skin barrier dysfunction [55]. IL-4 and IL-13, although classically linked to Th2 responses, have also been detected in rosacea lesions and contribute to chronic inflammation and fibrosis. Both cytokines activate STAT3 signaling, supporting alternative immune activation and tissue remodeling [56,57]. IL-31, a pruritogenic cytokine, signals through the IL-31 receptor complex to activate STAT3 and is strongly associated with chronic itch and neuroimmune sensitization [58,59]. These findings suggest that even low-level Th2 cytokine expression may contribute to the inflammatory phenotype of rosacea through STAT3-mediated mechanisms. In this study, topical CBG application significantly reduced the expression of pro-inflammatory cytokines (*Il1b*, *Il4*, *Il6*, *Il13*, *Il22*, and *Il31*) and downregulated *Tlr2*, a key upstream receptor in LL-37-mediated inflammation.

Taken together, our findings indicate that topical application of CBG attenuates multiple features of rosacea-like inflammation, including erythema, epidermal hyperplasia, immune cell infiltration, and abnormal vascular responses. These therapeutic effects appear to involve modulation of key inflammatory and angiogenic pathways, such as the YAP/TAZ and JAK/STAT signaling cascades. Notably, the lower dose (0.01 mg/kg) demonstrated greater efficacy than the higher dose, although the mechanism behind this inverse dose–response remains to be clarified. While the LL-37-induced model effectively mimics key inflammatory and vascular features of rosacea, it reflects an acute response and does not fully represent the chronic or subtype-specific nature of the human condition. In addition, our use of male mice follows standard practice in this model, though sex-related differences in rosacea warrant further investigation. These limitations highlight the need for additional validation in chronic or clinical settings.

It is also important to consider the evolving regulatory environment surrounding cannabis-derived compounds. Although CBG itself is non-psychoactive, most global regulations including those in Korea, the United States, and the European Union still impose strict limitations based on tetrahydrocannabinol (THC) content. Current guidelines generally permit research and topical applications when the THC level is below 0.2–0.3%, but CBG-specific classifications remain unclear [60,61]. As interest in non-psychoactive cannabinoids continues to grow, regulatory refinement will be essential for their broader clinical translation.

In addition to its potential as a standalone agent, evaluating the use of CBG in combination with existing rosacea therapies may represent an important future direction. Exploring such combinatorial approaches could enhance therapeutic efficacy and offer new insights into potential synergistic effects.

Although the development of CBG-based therapeutics is still in its early stages, our results suggest its strong anti-inflammatory and vascular-modulating potential. With expanding interest and gradually shifting regulatory frameworks, CBG may offer valuable opportunities in both clinical and industrial settings. These findings emphasize the therapeutic relevance of our experimental data, supporting the future clinical applicability of CBG as a novel, multi-targeted, and non-psychoactive treatment strategy.

## 4. Materials and Methods

Experimental procedures were adapted from previously established protocols developed in our laboratory [13,20,44]. Details of the primers and antibodies used in this study are summarized in Table 1 and Table 2.

### 4.1. Animal Study

This experiment employed male C57BL/6 mice aged six weeks at the time of study initiation (SLC, Inc., Shizuoka, Japan). A total of 40 mice were included in this study. The animals were acclimatized for 1 week before the experiment. During the experimental period, the mice were housed under controlled environmental conditions (22 °C, 12 h light/dark cycle) with free access to food and water.

LL-37 (320 μM) was injected subcutaneously four times at 12 h intervals over two days. The LL-37-induced rosacea-like mouse model was established as previously described [62]. Three days prior to LL-37 injection, the dorsal regions of the mice were shaved using an animal clipper and depilatory cream. A total of 50 μL of 320 μM LL-37 (tlrl-l37, InvivoGen, San Diego, CA, USA), dissolved in nuclease-free water, was intradermally injected into the shaved dorsal skin every 12 h for two consecutive days.

The mice were arbitrarily allocated to five groups: Control, LL-37, LL-37 + Bri, LL-37 + 0.01 mg/kg CBG, and LL-37 + 0.1 mg/kg CBG. Each group consisted of eight mice (*n* = 8), which was determined based on both experimental feasibility and reference to previous studies using similar rosacea-like mouse models [63,64]. In particular, this sample size ensured statistical validity and sufficient tissue acquisition for multiple downstream analyses, including histology, qRT-PCR, Western blotting, and IHC. Control mice received nuclease-free water instead of LL-37. Immediately after each injection, the assigned topical treatment (vehicle, brimonidine, or CBG) was applied to the injection site. Topical application was performed using a uniform volume of 20 μL across all groups to ensure consistency. CBG-treated groups received 20 μL of CBG solution prepared in a vehicle composed of 50% dimethyl sulfoxide (DMSO) and 50% distilled water (DW), adjusted to achieve the desired dose (0.01 or 0.1 mg/kg) based on mouse body weight. The LL-37 group received 20 μL of vehicle alone (without CBG), and the brimonidine group received 20 μL of unmodified brimonidine ophthalmic solution. For the Bri group, a commercially available ophthalmic solution, Alphagan^®^ P (0.15% brimonidine tartrate; AbbVie Inc., North Chicago, IL, USA), was used without modification.

Although a separate DMSO-only control group was not included, the LL-37-only group received the same DMSO-based vehicle used in the treatment groups, thereby ensuring consistency across experimental conditions. Previous studies have demonstrated that ≤0.1% DMSO does not impair cell viability or induce cutaneous toxicity and no visible irritation or adverse effects were observed during our study [65,66,67]. Nonetheless, in response to the reviewer’s comment, we acknowledge this as a study limitation and have added a corresponding note in the revised manuscript.

Twelve hours subsequent to the final injection, dermoscopic images of the dorsal skin were taken. Redness score was independently scored by three clinicians. After euthanasia, lesional skin samples were collected using 8 mm biopsy punches (Kai Medical, Kai Industries Co., Ltd., Seki City, Gifu, Japan) and were used for RNA extraction, hematoxylin and eosin (H&E) staining, and protein analysis. Redness score was evaluated on a scale ranging from 0 to 4, where 0 denoted the absence of symptoms, 1 indicated slight, 2 indicated moderate, 3 indicated severe, and 4 indicated very severe [68]. Redness area was quantified using ImageJ Fiji software (version 1.54g; Wayne Rasband and contributors, National Institutes of Health, Bethesda, MD, USA; https://imagej.net/downloads (accessed on 30 May 2025)). In order to minimize animal discomfort throughout the course of the procedures, such as shaving, drug administration, and clinical evaluation, isoflurane anesthesia (Ifran Solution, Hana Pharm. Co., Ltd., Seoul, Republic of Korea) was implemented.

All procedures of animal research were provided in accordance with the Laboratory Animals Welfare Act, the Guide for the Care and Use of Laboratory Animals, and the Guidelines and Policies for Rodent experiment provided by the IACUC (Institutional Animal Care and Use Committee) in school of medicine, The Catholic University of Korea. (Approval number: CUMS-2024-0026-02).

### 4.2. Histological Analysis

Skin tissues were preserved in 4% formaldehyde, processed for paraffin embedding, and sliced into 4 μm thick sections. For the purpose of histological evaluation, the sections were subjected to hematoxylin and eosin staining, while toluidine blue staining was employed to visualize mast cells. The number of mast cells was quantified from stained sections, and images were obtained using a DM2500 LED microscope (Leica Microsystems, Wetzlar, Germany). Epidermis thickness, as an indicator of epidermal hyperplasia, was measured using Leica Application Suite X software (version 3.7.1.21655; Leica Microsystems, Wetzlar, Germany).

### 4.3. Quantitative Real-Time PCR (qPCR) Analysis

To facilitate the extraction of ribonucleic acid (RNA), tissue samples were homogenized in TRIzol reagent (Invitrogen, Carlsbad, CA, USA). Subsequent to the process of homogenization, chloroform was introduced into the lysate and thoroughly integrated. Subsequently, the mixture was subjected to an incubation process at ambient temperature for a duration of 15 min. Thereafter, the mixture was subjected to a centrifugation process at a speed of 13,000 rpm for a duration of 15 min at a temperature of 4 °C. Subsequent to centrifugation, the upper aqueous phase containing RNA was meticulously separated and collected for further purification and analysis. The quality and concentration of the extracted RNA were assessed using a NanoDrop spectrophotometer (Thermo Fisher Scientific, Waltham, MA, USA). Following the quantification of RNA, diluted synthesized primers were combined with Power SYBR^®^ Green PCR Master Mix (Takara Biomedical Inc., Shiga, Japan). Quantitative real-time PCR was then performed using a CFX96 thermocycler (Bio-Rad Laboratories, Hercules, CA, USA). The protocol for the polymerase chain reaction (PCR) consisted of an initial 10 min denaturation period at 95 °C. This was followed by 45 cycles of 15 s denaturation at 95 °C and 30 s annealing/elongation periods at 60 °C. The protocol involved the application of PCR conditions and the 2−ΔΔCT method of analysis, as previously described [13,69]. Gene expression was normalized to *Actb*. The sequences of the primers utilized in this study are enumerated in Table 1.

### 4.4. Immunohistochemical Analysis

The process of immunohistochemical staining was initiated with the deparaffinization and rehydration of formaldehyde-fixed tissue sections. Antigen retrieval was carried out using preheated citrate buffer (pH 6.0; Agilent Technologies, Inc., Santa Clara, CA, USA), followed by incubation in a peroxidase-blocking solution (Agilent Technologies, Inc., Santa Clara, CA, USA) to inhibit endogenous enzyme activity. Subsequently, the slides were subjected to an incubation with primary antibodies (listed in Table 2). This incubation was conducted either overnight at 4 °C in a humid chamber or for a specified duration, as indicated in the relevant experimental section. Detection of horseradish peroxidase (HRP)-conjugated secondary antibodies was performed using the Dako REAL™ EnVision/HRP system (Agilent Technologies, Inc., Santa Clara, CA, USA) at room temperature. Visualization was achieved by applying a substrate–chromogen solution, and counterstaining was performed with Mayer’s hematoxylin (Agilent Technologies, Inc., Santa Clara, CA, USA). All measurements were performed using ImageJ Fiji software (version 1.54g; Wayne Rasband and contributors, National Institutes of Health, Bethesda, MD, USA; https://imagej.net/downloads (accessed on 30 May 2025)).

### 4.5. Western Blot Analysis

Protein lysates were prepared from dorsal skin tissues using T-PER lysis buffer supplemented with a protease inhibitor cocktail (Thermo Fisher Scientific, Waltham, MA, USA). Protein concentration was determined using the BCA Protein Assay Kit II (Thermo Fisher Scientific, Waltham, MA, USA). Equal amounts of protein (20 μg) were separated on 6–10% SDS-polyacrylamide gels and transferred onto polyvinylidene fluoride (PVDF) membranes (MilliporeSigma, St. Louis, MO, USA).

The membranes were blocked for 2 h at room temperature with either 5% non-fat dry milk or 5% bovine serum albumin (BSA) in Tris-buffered saline containing 0.1% Tween 20 (TBS-T). Primary antibodies (listed in Table 2) were incubated overnight at 4 °C. Following a series of four washes with TBS-T, the membranes were subjected to an incubation period of two hours at room temperature in the presence of horseradish peroxidase (HRP)-conjugated goat anti-mouse or anti-rabbit IgG secondary antibodies (listed in Table 2).

The visualization of protein bands was accomplished through the utilization of an enhanced chemiluminescence (ECL) substrate (Thermo Fisher Scientific, Waltham, MA, USA) followed by detection with the Amersham™ Imager 600 system (GE Healthcare, Chicago, IL, USA). The quantification of band intensities was performed using ImageJ software (version 1.54f; Wayne Rasband and contributors, National Institutes of Health, Bethesda, MD, USA; https://imagej.net/downloads (accessed on 30 May 2025)).

### 4.6. Statistical Analysis

Statistical analysis was performed using one-way ANOVA, followed by Tukey’s multiple comparison test to evaluate differences among groups. Pairwise comparisons were assessed using unpaired *t*-tests. Graphs were generated with GraphPad Prism software (version 5; GraphPad Software Inc., La Jolla, CA, USA). All results are presented as mean ± standard error of the mean (SEM). A *p*-value below 0.05 was considered statistically significant (* *p* < 0.05, ** *p* < 0.01, *** *p* < 0.001).

## 5. Conclusions

**Figure 5:**
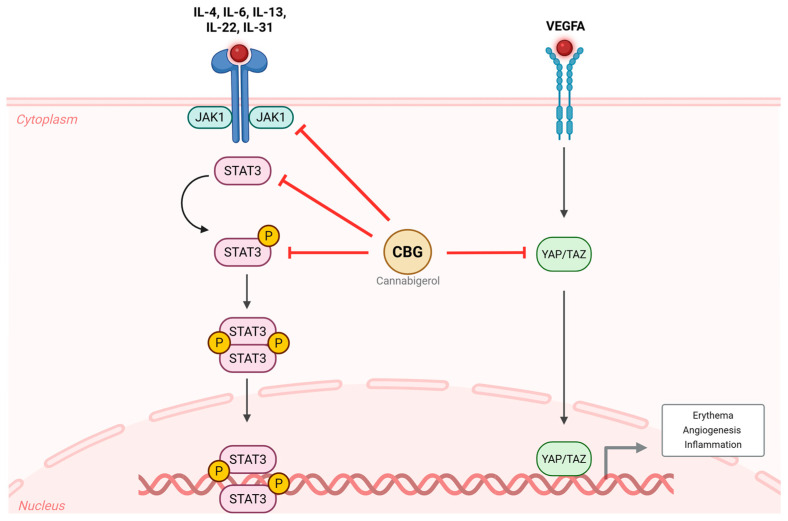
Effects of CBG on inflammatory and vascular signaling pathways in rosacea. In the LL-37-induced rosacea-like mouse model, inflammatory cytokines (*Il4*, *Il6*, *Il13*, *Il22*, *Il31*) activate the JAK1/STAT3 signaling pathway, and *Vegfa* promotes YAP/TAZ activation. These pathways act in concert to drive erythema, angiogenesis, and inflammation. Topical treatment with CBG inhibited the phosphorylation of STAT3 and suppressed YAP/TAZ activity, thereby attenuating rosacea-related inflammatory and vascular responses. CBG, cannabigerol; VEGFA, vascular endothelial growth factor A; JAK, Janus kinase; STAT, signal transducer and activator of transcription; YAP, yes-associated protein; TAZ, transcriptional coactivator with PDZ-binding motif; IL, interleukin.

In conclusion, our study demonstrated the therapeutic potential of CBG in an LL-37-induced mouse model of rosacea. Topical CBG treatment significantly reduced clinical erythema, epidermal hyperplasia, and mast cell infiltration, and suppressed key inflammatory and vascular mediators at both the mRNA and protein levels. Mechanistically, CBG inhibited the expression of cytokines, *Vegfa*, and *Tlr2*, as well as the activation of YAP/TAZ and JAK/STAT signaling pathways, which are known to be involved in rosacea pathogenesis (Figure 5). These findings highlight CBG as a promising non-psychoactive cannabinoid with therapeutic relevance for the treatment of rosacea.

## Figures and Tables

**Table 1:** Primer sets employed in quantitative real-time PCR.

Target	Sequence (5′-3′)	
*Mmp9*	Forward	GCCCGGAACTCACACGACA
	Reverse	TTGGAAACTCACACGCGAGAAG
*Tlr2*	Forward	CTCTTCAGCAAACGCTGTTCT
	Reverse	GGCGTCTCCCTCTATTGTATTG
*Vegfa*	Forward	TATTCAGCGGACTCACCAGC
	Reverse	AACCAACCTCCTCAAACCGT
*Il1b*	Forward	TGC CAC CTT TTG ACA GTG AT
	Reverse	AGT GAT ACT GCC TGC CTG AA
*Il4*	Forward	TCTCGAATGTACCAGGAGCCATATC
	Reverse	AGCACCTTGGAAGCCTACAGA
*Il6*	Forward	CCC CAA TTT CCA ATG CTC TCC
	Reverse	AGG CAT AAC GCA CTA GGT TT
*Il13*	Forward	CTGCTACCTCACTGTAGCCT
	Reverse	TATTTCATGGCTGAGGGCTG
*Il22*	Forward	TTCCGAGGAGTCAGTGCTAA
	Reverse	GAGTTTGGTCAGGAAAGGCA
*Il31*	Forward	ATACAGCTGCCGTGTTTCAG
	Reverse	AGCCATCTTATCACCCAAGAA
*Actb*	Forward	TGTGATGGTGGGAATGGGTCAGAA
	Reverse	TGTGGTGCCAGATCTTCTCCATGT

**Table 2:** List of primary antibodies applied for IHC and Western blot analyses.

Assay	Antibody	Dilution	Cat No.	Source
WB	β-actin	1:2500	#3700	Cell Signaling Technology^®^, Danvers, MA, USA
WB/IHC	CD31	1:1000/ 1:200	#77699	Cell Signaling Technology^®^
WB/IHC	VEGF	1:1000/1:500	AB416154	Abcam, Cambridge, UK
WB/IHC	YAP	1:500/1:100	sc-376830	Santa Cruz, Dallas, TX, USA
WB/IHC	TAZ	1:500/1:50	sc-518026	Santa Cruz
WB/IHC	JAK1	1:400/1:100	#3344	Cell Signaling Technology^®^
WB/IHC	STAT3	1:1000/1:500	#9139	Cell Signaling Technology^®^
WB/IHC	p-STAT3	1:1000/1:200	#9145	Cell Signaling Technology^®^
IHC	Filaggrin	1:500	905804	BioLegend, San Diego, CA, USA
IHC	Vimentin	1:500	AB92547	Abcam
IHC	α-SMA	1:500	14-9760-82	Invitrogen
WB	Goat Anti-Mouse IgG antibody (HRP)	1:4000	GRX213111-01	GeneTex, Irvine, CA, USA
WB	Goat Anti-Rabbit IgG antibody (HRP)	1:4000	GRX213110-01	GeneTex

Abbreviations: WB; Western blotting; IHC, Immunohistochemical.

## Data Availability

The data are included in the article.
